# Clinical and Pharmacologic Differences of CDK4/6 Inhibitors in Breast Cancer

**DOI:** 10.3389/fonc.2021.693104

**Published:** 2021-07-12

**Authors:** Mridula A. George, Sadaf Qureshi, Coral Omene, Deborah L. Toppmeyer, Shridar Ganesan

**Affiliations:** ^1^ Rutgers Cancer Institute of New Jersey, Rutgers, The State University of New Jersey, New Brunswick, NJ, United States; ^2^ Rutgers Robert Wood Johnson Medical School, Rutgers, The State University of New Jersey, New Brunswick, NJ, United States

**Keywords:** metastatic breast cancer, CDK4/6 cell cycle inhibitors, hormone receptor (HR), palbociblib, ribociclib, abemaciclib, CDK4/6 inhibitors in breast cancer

## Abstract

Targeted therapies such as Cyclin Dependent Kinase 4 and 6 (CDK 4/6) inhibitors have improved the prognosis of metastatic hormone receptor (HR) positive breast cancer by combating the resistance seen with traditional endocrine therapy. The three approved agents currently in the market are palbociclib, ribociclib and abemaciclib. Besides the overall similarities associated with CDK4/6 inhibition, there are differences between the three approved agents that may explain the differences noted in unique clinical scenarios- monotherapy, patients with brain metastases or use in the adjuvant setting. This review article will explore the preclinical and pharmacological differences between the three agents and help understand the benefits seen with these agents in certain subgroups of patients with metastatic HR positive breast cancer.

## Introduction

The approval of Cyclin Dependent Kinase 4 and 6 (CDK 4/6) inhibitors changed the treatment landscape for patients with hormone receptor (HR) positive, human epidermal growth factor receptor 2 (HER2)-negative advanced breast cancer. Currently approved agents include palbociclib, ribociclib and abemaciclib which are all approved for concurrent use with hormonal therapy based on the randomized phase III trials PALOMA, MONALEESA and MONARCH studies (1–7) respectively. These agents have significantly improved progression free survival when combined with anti-estrogen therapy compared to monotherapy with anti-estrogen therapy. Some agents have also shown statistically significant improvement in overall survival in the metastatic setting.

Although these agents have reported comparable improvements in progression free survival (PFS) in advanced/metastatic HR positive breast cancer when given in combination with hormonal therapy, there are unique differences between these agents in their pharmacology, kinase targets, central nervous system (CNS) penetration and clinical activity as single agents. These differences may contribute to the reported differences in activity of these agents in the adjuvant treatment of early-stage breast cancer. This article will review preclinical and biological differences among the three FDA approved CDK 4/6 inhibitors that can help understand the differences in their clinical activity.

## Cell Cycle Dysregulation

Cyclins and CDKs are essential in regulating the progression through the distinct phases of the cell cycle, G1, S, G2 and M phases and hence play an important role in regulating cell cycle transitions. CDKs are serine/threonine kinases which are regulated by their interactions with cyclins and CDK inhibitors. CDK activity is often dysregulated in cancer cells and hence they are an attractive target for anti-cancer therapy. In human cells, there are 20 CDKs and 29 cyclins (8). CDK1, CDK2, CDK3, CDK4, CDK6, and CDK7 directly regulate cell-cycle transitions and cell division, whereas CDK 7–11 mediate cell-cycle associated gene transcription (9–12).

**Figure 1:**
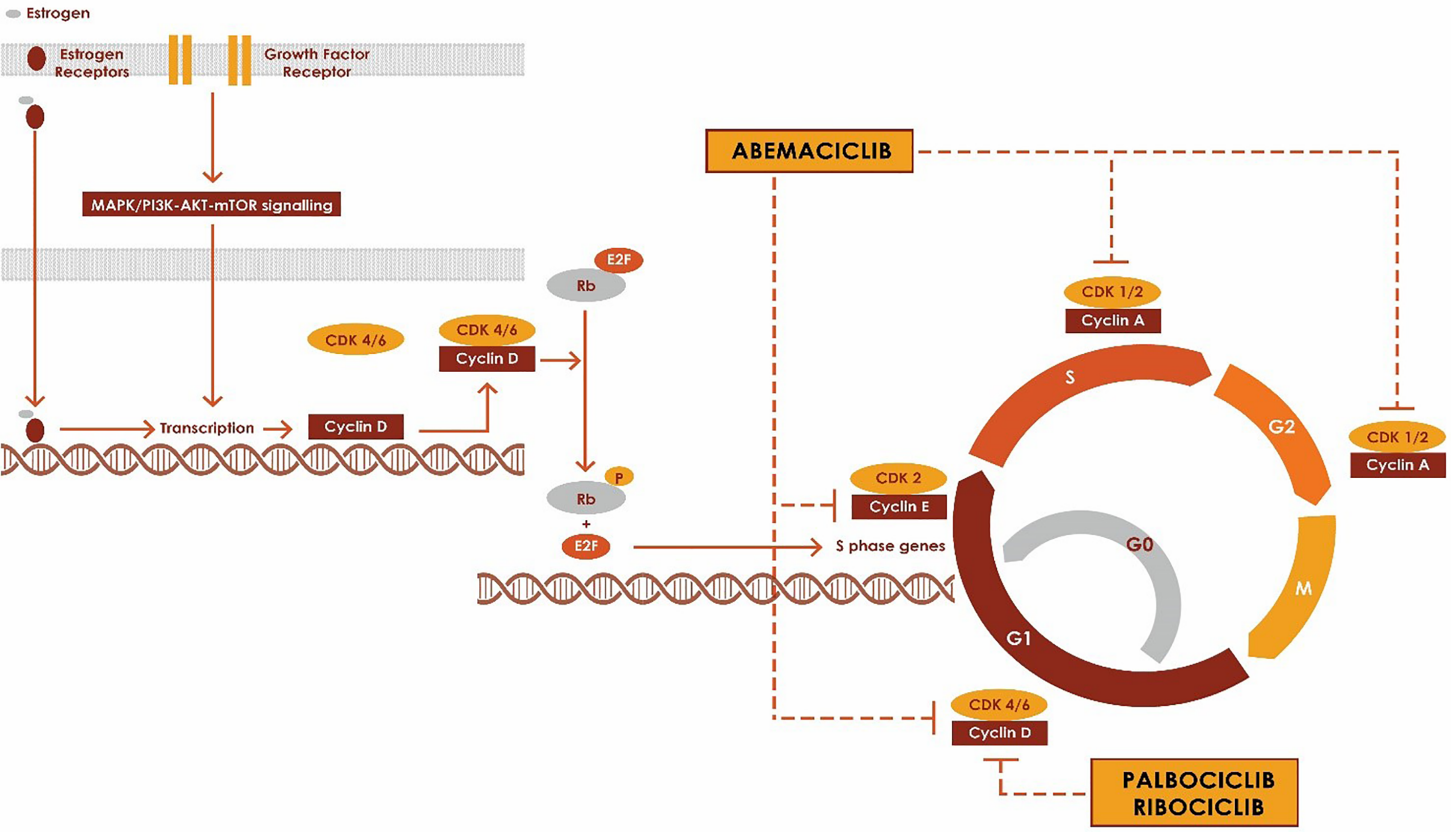
Mechanism of action of CDK 4/6 inhibitors.

Mitogenic signals from receptor tyrosine kinases and downstream signaling events such as RAS, phosphatidylinositol 3-kinase (PI3K), mitogen-activated protein kinase (MAPK), mammalian target of rapamycin (mTOR) and nuclear receptors (estrogen receptor, progesterone receptor and androgen receptor) drive the progression of the quiescent cells from G0 or G1 phase into S phase through CDK4 or CDK6 complex (10, 13, 14). CDK4 and CDK6 primarily associate with D-type cyclins (Cyclin D1, Cyclin D2 and cyclin D3) to form Cyclin-CDK complexes which regulate the progression in the cell cycle *via* phosphorylation of tumor suppressor protein retinoblastoma (15). (**Figure 1**) The phosphorylation of Rb disrupts the binding of Rb to E2F transcription factors, freeing E2F and initiating the progression of the cell through the cell cycle by the expression of genes such as cyclin E (16). CDK4/6 inhibitors block the cell cycle transition from G1 to S by inhibiting the kinase activity of the CDK/cyclin complex and hence preventing phosphorylation of Rb protein, which is a key step in the progression into the cell cycle. RB1 has to be intact for CKD4/6 inhibitors to impact cell cycle progression, and RB1-mutant cancers are resistant to CKD4/6 inhibitors. In ER+ breast cancer, they work synergistically with anti-estrogen therapies such as aromatase inhibitors, Fulvestrant and tamoxifen (1, 2, 4, 7, 17, 18).

Increased activity through mitogenic signaling pathways such as PI3K, mTOR, steroid receptor pathways also results in activation of cyclinD-CDK 4/6 activity. Cyclin D1 is a direct transcriptional target of ER, and estrogens promote the transit of ER-positive breast cancer cells through the cell cycle (19). Cyclin D1 can be activated through pathways dependent and independent of estrogen (20). Over-expression of cyclin D1 increases the activity of CDK4/6 (21, 22). Cyclin/CDKs are negatively regulated by cyclin-dependent kinase inhibitors (CKIs) such as p16^INK4A^, p15^INK4B^, p18^INK4C^ and p19^INK4D^ which primarily inhibit CDK4 and CDK6. Deletions resulting in the loss of p16^INK4A^ or Rb expression also result in tumorigenesis (23, 24). Cyclin D‐CDK4/6–INK4–Rb pathway regulates the progression of the breast cancer cell through G1–S phases of the cell cycle.

Besides the overall similarity in terms of class activity of the approved CDK4/6 inhibitors, there are subtle differences among these agents, including differences in substrate selectivity and pharmacodynamics that can explain the varying differences seen in certain clinical settings, and will be reviewed below. No head-to-head studies have compared the approved CDK4/6 inhibitors.

### Pharmacology

Although all CDK4/6 inhibitors share the same primary mechanism of action, they have considerable pharmacologic differences. Ribociclib and palbociclib have greater lipophilicity and larger binding site side chains when compared to abemaciclib (25). Abemaciclib forms a hydrogen bond to the ATP cleft with a catalytic residue that is common amongst many kinases. This is not seen in palbociclib and ribociclib. Another difference in drug binding is that abemaciclib buries two fluorine atoms against the back wall of the ATP-binding pocket, whereas palbociclib and ribociclib present much larger substituents (ribociclib’s dimethylamino group; palbociclib’s methylketone and adjacent methyl) that might be more difficult to accommodate in other kinases (26). These differences may mediate selectivity of an inhibitor with off-target kinases such as other CDK family members (26). The CDK-6-Abemaciclib complex is noted to have a water molecule bridging histidine-100 residue on the binding site and the ligand, which is not observed with palbociclib and ribociclib (26). Histidine100 is found only in CDK 4/6 and the bridging allows for favorable kinase selectivity (26).

All three CDK4/6 inhibitors undergo hepatic metabolism by cytochrome P450 3A4 (CYP3A4). Palbociclib is metabolized by both CYP3A4 and sulfotransferase enzyme (SULT2A1). Palbociclib should be dose-reduced to 75 mg daily if concomitant use of a strong CYP3A4 inhibitor is required. No dosage adjustments are required for mild or moderate hepatic impairment (Child-Pugh classes A and B) for any of the CDK4/6 inhibitors. However, for severe (Child-Pugh class C) hepatic impairment, dose adjustments are recommended for all three agents; Palbociclib should be reduced to 75mg daily, abemaciclib should be 200 mg daily (rather than twice per day), and ribociclib should be reduced to 400 mg daily (25, 27).

No dose adjustments are required for impaired renal function with any of the CDK-4/6 inhibitors (28).

In a phase 1 study of the bioavailability of palbociclib, food intake modestly increased drug absorption and decreased variability in serum drug concentration over time, as determined by the area under the curve (29). Food has not been shown to affect the bioavailability of either ribociclib or abemaciclib.

The mean half-life of palbociclib is 29 (+/- 5) hours (30), ribociclib is 32 hours (31) and abemaciclib is18.3 hours (32). Palbociclib and ribociclib have a longer half-life than abemaciclib and hence are administered once a day. Abemaciclib needs twice daily administration. (See **Table 1**) The difference in dosing schedules affect the serum plasma concentrations of the drugs. The longer, > 24-hour half-lives of palbociclib and ribociclib in conjunction with daily dosing may result in drug accumulation. As a result, palbociclib and ribociclib have non-continuous dosing requiring a one week break between cycles.

**Table 1:** Pharmacology of the three approved CDK4/6 inhibitors.

	Palbociclib	Ribociclib	Abemaciclib
**Half-life**	29 (+/-5) hours	32 hours	18.3 hours
**Primary site of metabolism**	Hepatic	Hepatic	Hepatic
**Cell Cycle Arrest**	G1 phase	G1 Phase	G1, G2 phase
**Targets**	CDK4 and CDK6	CDK4 and CDK6	CDK1, CDK2, CDK4, CDK5 CDK6, CDK 9, CDK14, CDKs16-18
**Dosing**	125mg once daily for 21 days followed by 7 days off	600mg one daily for 21 days	150mg twice day continuously
**Myelosuppression**	++	++	+
**GI toxicity**	+	+	++
**LFT abnormalities**	–	+	+
**Pneumonitis**	+ (rare)	+ (rare)	+ (rare)

### Cyclin Dependent Kinase Selectivity

Palbociclib is a reversible small molecule CDK 4/6 inhibitor which is highly specific for CDK 4 and CDK 6 (33). Palbociclib and ribociclib are based off of a similar pyrido [2,3-d]pyrimidin-7-one scaffold that was optimized for selectivity toward CDK4/6 (16, 34). Similarly, abemaciclib, is a reversible inhibitor but developed from a 2-anilino-2,4-pyrimidine-[5-benzimidazole] scaffold (16, 35).

All three drugs inhibit CDK 4 and CDK 6 kinase activity, however abemeciclib inhibits multiple other kinases as well (35). Palbociclib has similar potency against cyclin D1/CDK4 and cyclin D2/CDK6 (36). Abemaciclib and ribociclib were noted to have greater potency against CDK4 than CDK 6 (35, 37). Abemaciclib is 14 times more potent against CDK4 than it is against CDK6 (38). Abemaciclib has five-fold more potency for CDK4 than palbociclib or ribociclib (16, 35). Unlike palbociclib and ribociclib, abemaciclib has been shown to have in-vivo inhibition of CDK1, CDK2, CDK5, CDK9, CDK14, CDKs16-18, GSK3α/β, CAMKIIγ/δ and PIM1 kinases (35, 39). Abemaciclib is 10- to 100-fold less potent against CDK2 and CDK1 than CDK4/6 (40).

CDK4/6 inhibitors induce cytostasis through cell-cycle arrest in the G1 phase, resulting in growth inhibition. However, pre-clinical models of abemaciclib demonstrated that it can induce tumor cell death and regression, rather than cell cycle arrest alone. This is evidenced by the fact that monotherapy with abemaciclib in a Phase I study was shown to decrease tumor size, rather than simply inhibit growth in solid tumors including breast cancer (41). Based on these results, abemaciclib was thought to have additional mechanisms of action outside of just inducing G1-cell cycle arrest.

Hafner et al. evaluated the phenotypic and biochemical difference by studying the transcriptional, proteomic, and phenotypic changes between the three agents (40). Abemaciclib was seen to cause cell arrest in G2 phase as well as GI phase most likely due to the inhibition of CDK 1 and CDK2, which are required for progression through S phase and mitosis (40). This causes cells to accumulate in G2 phase consistent with a cell cycle arrest independent of CDK4/6, which was not seen with palbociclib and ribociclib (40).

Hafner et al. also studied the dose response curves in 34 breast cancer cell lines to evaluate cell-cycle arrest and cell death induced by CDK4/6 inhibitors. Abemaciclib was found to be 5.5 times more potent at inducing cytostasis compared to palbociclib based on GR50 values (the dose required to decrease cell growth by 50%). At higher doses, abemaciclib is cytotoxic, even in the absence of Rb. The Rb-independent effects of abemaciclib suggest it is acting on other targets besides CDK4/6. However, palbociclib and ribociclib caused cytostasis or cell cycle arrest, at all dose levels. There was no cytotoxicity seen with either drug (40).

Cells adapt to CDK4/6 inhibition by developing mechanisms of resistance, including acquired mutations in RB1 (42). Palbociclib-adapted cells were also resistant to ribociclib, however they responded to abemaciclib (40). In a patient cell line established from a patient with metastatic breast cancer with progression of disease after ribociclib/letrozole, abemaciclib induced cell death and inhibited cell proliferation. The cell line was noted to be RB deficient suggesting again that clinical activity of abemaciclib may be in part due to targets other than CDK4/6 (40).

Multiplexed inhibitor bead mass spectrometry (MIB/MS) platform was used to compare the kinase targets of palbociclib and abemaciclib (43). MIB/MS profiling showed that unlike palbociclib, abemaciclib activates wnt signaling *via* inhibition of glycogen synthase kinase GSK3β and subsequent stabilization of β-catenin (43). GSK3β activity also plays an important role in the regulation of cyclin D family proteins at both the transcriptional and proteomic level such that inhibition of GSK3β is expected to increase the levels of cyclin D (43). Palbociclib and ribociclib do not impact GSK3β or WNT signaling (43).

### Dosing and Side Effect Profile

The CDK 4/6 inhibitors fall into two broad categories based on their toxicity profile and the dose –delivery schedules (44). Palbociclib and ribociclib both have >24-hour half-lives and are dosed daily. Abemaciclib has a shorter half-life and is dosed twice daily. Due to their myelosuppressive effects, Palbociclib and ribociclib are dosed daily for 21 days followed by a one- week break to enable neutrophil count recovery in patients. Abemaciclib is dosed continuously without a break and is associated with lower incidence and less severe bone marrow suppression compared to the other agents (See **Table 1**).

Even though all three drugs have a similar mechanism of action, they have varying side effect profiles. The differential targets of these drugs, especially the relative potency of CDK4 vs CDK6 and are most likely responsible for the different dose limiting toxicities (45). Some of the pertinent side effects for each of the drugs are as follows: Neutropenia, leukopenia and fatigue for palbociclib; neutropenia, increased creatinine, hyponatremia, QTc prolongation for ribociclib; diarrhea and fatigue for abemaciclib.

All three agents have a FDA warning about rare but serious case of pneumonitis. Pneumonitis was reported for 9 (2.0%) and 17 (5.2%) abemaciclib‐treated patients in MONARCH 2 and 3, respectively, with ≤1% of cases grade ≥3 (46). In both studies, patients who had pneumonitis had history of prior radiation or lung metastases.

Bone marrow toxicity associated with abemaciclib is not as severe as the bone marrow toxicity associated with palbociclib or ribociclib. Palbociclib and ribociclib have higher rates of hematologic toxicity, primarily neutropenia. Although the risk of neutropenia was higher, the risk of neutropenic fevers were rare (47). In the pooled safety analysis of the three randomized phase II and III trials of Palbociclib, the rates of grade III and IV neutropenia were 55.3% and 10.1% in the treatment arm (48). Relatively few patients experienced febrile neutropenia (1%), and the rate of permanent treatment discontinuation associated with neutropenia were low (0.7%) (48). In the pooled safety analysis of Ribociclib across the three studies (MONALEESA-2, MONALEESA- 3 and MONALEESA-7), neutropenia and leukopenia were the most common cause of grade 3/4 adverse event (49). In the pooled analysis of MONARCH 2 and MONARCH 3, the rate of grade 3 and Grade 4 neutropenia associated with abemaciclib were 22.9% and 2.5% respectively (46). Dose reductions owing to neutropenia were 10-13% in all patients treated with abemaciclib and discontinuation in 1-3% (46). Given the risk of neutropenia, FDA mandates checking blood counts every 2 weeks for the first 2 months, monthly for the next 2 months and as clinically indicated.

With regards to GI toxicity, palbociclib and ribociclib have minimal GI toxicities in contrast to abemaciclib, which has pervasive GI toxicities such as nausea, vomiting and diarrhea. In the safety analysis of MONARCH-2, and MONARCH-3, diarrhea was the most frequently reported AE occurring in 85% of patients (46). 10-13% of patients reported grade 3 diarrhea and no patients reported grade≥4 diarrhea (46). The median time to onset for any-grade diarrhea was 6 days in MONARCH 2 and 8 days in MONARCH 3 (46). The high rates of clinically significant diarrhea was observed in the first cycles on MONARCH-2 and MONARCH- 3, with decreasing incidence in subsequent cycles (46). Dose reductions due to diarrhea occurred in 13-19% of patients and discontinuations due to diarrhea occurred in 2.3% to 2.9% in MONARCH 2 and MONARCH 3, respectively (46). Dose reductions and anti-diarrheal medications are used to manage diarrhea. The rates of diarrhea observed with palbociclib were comparable to the control arm (48).

In a systematic review evaluating the toxicities of the three approved drugs, ribociclib was noted to have higher rates of hepatic toxicity in a systematic review evaluating the adverse events associated with all three agents (50). These side effects were dose-dependent and reversible with drug withdrawal (50). Ribociclib was noted to have any- grade ALT and AST increases in 13.8% and 12.7% of patients in pooled safety analysis of MONALEESA-2, MONALEESA- 3 and MONALEESA-7 (49). In the pooled safety analysis of MONARCH-2 and MONARCH-3 trial, the rates of any grade ALT and AST rise was 15.1% and 14.2% respectively (46). Livers tests need to be monitored for patients on ribociclib and abemaciclib, at baseline and every 2 weeks for 2 months, then monthly for the next 2 months and as clinically indicated.

In the pooled safety analysis of MONALEESA 2,3 and 7, 69 patients (n=1065) in the treatment arm had prolonged QT compared to 13 patients (n=818) in the placebo group (49). Fridericia’s corrected QT interval (QTcF) >480 ms occurred in 5% of patients in the treatment arm compared to 1% of pts in the placebo arm (49). Given the risk of QTc prolongation, ribociclib is recommended only in patients with QTcF<450msec. Baseline EKG is recommended for patients on ribociclib at baseline, day 14 of cycle 1 and day 1 of cycle 2. Palbociclib was shown to have no clinically relevant effect on QTc prolongation (51). QT prolongation was not reported as an adverse event in the pooled analysis of MONARCH 2 and MONARCH 3 (46).

### Clinical Activity

#### Monotherapy

A single arm phase II study evaluated palbociclib monotherapy in patients with metastatic breast cancer that was positive for Rb expression (all subtypes were eligible) (52). 90% of the patients had received prior chemotherapy and 78% had received prior hormonal therapy. The clinical benefit rate was 21% (7% PR, 14% SD>6months). The median PFS was 4.1 months (95% CI 2.3–7.7) for patients with ER+ HER2- metastatic breast cancer, 18.8 months (95% CI: 5.1—NE) for ER+ HER2+ patients and 1.8 months (95% CI: 0.9—NE) for patients with triple negative tumors, respectively (52).

The TREnd trial studied single agent activity of palbociclib in women with advanced breast cancer. Patients were randomized to receive palbociclib alone or in combination with ET (endocrine therapy). The CBR was similar in both arms, 54% (95% CI: 42–67%) in the combination one, and 60% (95% CI: 48–73%) with palbociclib alone (53). The trial was not powered to estimate survival endpoints, however exploratory analyses were performed, with no significant differences observed in PFS (*p* = 0.13) - median PFS was 10.88 months in the combination arm compared to 6.5 months with palbociclib alone. (Hazard ratio (HR) 0.69; 95% CI: 0.4–1.1, exploratory P-value = 0.12) (53).

Single agent data with Ribociclib is available in phase I studies which showed partial response in 1 patient with HR+ breast cancer (54) and stable disease was noted in another patient with heavily pre-treated breast cancer (55).

A phase I study evaluated abemaciclib in 47 patients with different subtypes of breast cancer: HR-positive (36), HR negative (9); HR-positive/HER2-positive (11) (56). The clinical benefit rate was higher in HR-positive subgroup compared to the HR-negative subgroup. The PFS was 8.8 months in HR positive patients compared to 1.1 months in HR negative subgroup (57). Abemaciclib was further studied as monotherapy in women HR+ HER2– metastatic breast cancer in the MONARCH-1 study. Patients who had progressed on prior ET and had received at least two prior chemotherapy regimens were eligible (58). The medial PFS was 6 months (95% CI 4.2–7.5). The ORR (primary endpoint) was 19.7% (95% CI, 13.3–27.5), the CBR was 42.4% (19% PR, 47.7% SD>6months), and median overall survival (OS) was 17.7 months (95% CI, 16 to not reached). Based on this study, the FDA approved abemaciclib as monotherapy in pretreated patients with HR+ HER2– metastatic breast cancer (58). Palbociclib and Ribociclib are currently not approved for monotherapy in the management of metastatic breast cancer.

#### Metastatic Setting

The combination of CDK4/6 inhibitors with endocrine therapy became standard of care in the metastatic setting largely based on 7 phase III studies. (See **Table 2**).

**Table 2:** Clinical Activity of the CDK4/6 inhibitors in different clinical settings.

	Palbociclib	Ribociclib	Abemaciclib
**Monotherapy**	**Not approved**	**Not approved**	**FDA approved for monotherapy**
**CNS activity**	**-**	**-**	**+**
**Adjuvant Setting**	**No Benefit** 3-y IDFS 88.2% vs. 88.5% HR 0.93; 95% CI 0.76-1.15 **(PALLAS)** 3y-IDFS 82.1% vs. 77.7% HR 0.93; 95% CI 0.74 to 1.17 p=0.525 **(PENELOPE-B)**	**Study Ongoing** **(NATALEE)**	**Shown to have benefit** 2y-IDFS 92.2% vs. 88.7% HR 0.75; 95% CI, 0.60 to 0.93, P = .01. **(MonarchE)**

The PALOMA-2 trial showed that palbociclib + letrozole improved PFS compared to letrozole alone (24.8 months vs. 14.5 months, respectively) (59). PALOMA-3 confirmed improvement in median PFS (9.5 months vs. 4.6months;HR 0·46, 0·36–0·59, p<0·0001). Ribociclib was FDA approved in 2017 for pre-, post-, and peri-menopausal women either in combination with AI or in combination with fulvestrant for postmenopausal women or those who have progressed on prior endocrine therapy. MONALEESA-2, MONALEESA-3 and MONALEESA-7 were all phase III studies which showed statistically significant improvement in PFS. Abemaciclib was FDA approved in 2017 based on the MONARCH-2 study which showed statistically significant improvement in progression free survival and overall survival. In a subset analysis, there was statistically significant improvement in time to second disease progression (median, 23.1 months vs 20.6 months), time to chemotherapy (median, 50.2 months vs 22.1 months), and chemotherapy-free survival (median, 25.5 months vs 18.2 months) (60).

Five studies evaluated CDK4/6 inhibitors in de-novo metastatic patients and showed improvement in PFS (PALOMA-2, MONALEESA-2, MONALEESA-3, MONARCH-3, MONALEESA-7). (see **Table 2**) Overall survival data for PALOMA-2 and MONARCH-3 have not been reported. MONALEESA-3 reported statistically significant improvements in OS. MONALEESA-7 was a phase III study in pre-menopausal women which also showed improvement in overall survival (61).

Three phase 3 studies evaluated CDK4/6 inhibitors in patients who progressed on prior endocrine therapy-PALOMA-3, MONALEESA-3 and MONARCH-2. Although all studies showed improvement in PFS, only MONALEESA-3 (HR 0.686; 0.451-1.041) and MONARCH -2 showed statistically significant improvement in OS. In PALOMA-3, the overall survival (OS) was not statistically significant (34.9months vs, 28months HR: 0.81; 95% CI, 0.64 to 1.03; P=0.09). PALOMA-3 enrolled patients who had progressed on more than one line of endocrine therapy. In women who had documented sensitivity to previous endocrine therapy, there was a statistically significant improvement in overall survival. (39.7 months vs. 29.7 months HR 0.72; 95% CI, 0.55 to 0.94)).

In patients with bone-only disease, there was a marked improvement in PFS in the palbociclib arm (in PALOMA-2 trial, 36.2 versus 11.2 months, p<0.0001; in PALOMA-3, 14.3 versus 9.2 months, p=0.0394) (62). In the PALOMA trials, patients with visceral metastasis had a greater PFS with palbociclib than in the control arms (PALOMA-2: 19.3 versus 12.9 months, p<0.005; PALOMA-3: 8.0 versus 3.5 months, p=0.82) although the magnitude of benefit appears less compared to bone-only disease (62). In the pooled data from MONALEESA-3 and MONALEESA-7, there was OS benefit in the patients who had visceral disease treated with ribociclib plus endocrine therapy compared to the placebo arm. Specifically in patients who had liver metastases, OS was 36.1 in ribociclib vs 24.1 months in the placebo arm (HR 0.629 95% CI, 0.421-0.942) and in MONALEESA-7 OS was NR vs. 33.6 months in patients who had liver metastases (HR 0.531; (95% CI, 0.321-0.877) (63) The OS benefit with abemaciclib was more pronounced in patients with visceral disease compared to bone-only disease.(Visceral disease HR, 0.675; 95% CI, 0.511-0.891) compared with bone-only disease (HR,0.907; 95% CI,0.564-1.457) (60) The PFS in patients with liver metastases treated with abemaciclb plus an AI was 15.0 months versus 7.2 months for placebo plus an AI, (HR, 0.477; 0.272-0.837). In patients with bone only disease, palbocilclib should be considered over the other agents. Abemacilib and ribociclib have greater activity in patients who have visceral involvement.

#### Adjuvant Setting

In the PALLAS study, the addition of 2 years of palbociclib or placebo to ET was evaluated in patients with HR+/HER2 negative Stage II-III breast cancer (64). At interim analysis, the 3-year IDFS was similar in both arms (88.2% for combination arm, and 88.5% for ET alone HR 0.93, 95% confidence interval 0.76-1.15), and crossed the pre-specified futility boundary (64). Similarly, PENELOPE-B, a phase III, double blind, placebo-controlled study that evaluated 1 year of palbociclib or placebo to ET in high risk patients with HR+/HER2 negative residual invasive disease following neoadjuvant chemotherapy. At median follow up of 42.8 months, there was no improvement in iDFS (HR 0.93; 95% CI 0.74 to 1.17 p=0.525) or overall survival benefit for palbociclib. (HR 0.87; 95% CI 0.61 to 1.22; p=0.420) (65).

The MonarchE study evaluated the addition of 2 years of abemaciclib or placebo to ET in patients with high-risk HR+/HER2 negative breast cancer (66). High risk was defined as four or more positive nodes, or one to three nodes and either tumor size ≥ 5 cm, histologic grade 3, or central Ki-67 ≥ 20%. At the interim analysis, abemaciclib plus ET demonstrated a statistically significant improvement in 2-year iDFS versus ET alone (92.2% vs. 88.7% P = .01 HR, 0.75; 95% CI, 0.60 to 0.93) (66). The combination had an improvement in distant recurrence free survival (93.6% with combination compared to 90.3 with ET alone p=0.01 HR, 0.72; 95% CI, 0.56 to 0.92) (66).

It is unclear if the difference seen in these adjuvant studies of palbociclib and abemaciclib was due to differences in the study population or due to intrinsic differences between the drugs. Longer follow up of MonarchE will be need to determine if the improved iDFS is robust and maintained. Ribociclib is currently being studied in the adjuvant setting in the NATALEE study. (ClinicalTrials NCT03701334). The study was amended to include high risk patients. (**Table 3**)

**Table 3:** Overview of the clinical trials for the approved CDK4/6 inhibitors in the metastatic setting.

Name of the studies	Phase	Line of therapy	Treatment arms	Sample Size	PFS	Median OS
**Palbociclib**						
PALOMA-1	II	First line	Palbociclib/Letrozole vs. Letrozole	165	20.2months vs. 10.2 months (HR 0.488, 0.319-0.748; p=0.0004) (66)	Not significant 37.5 vs 34.5 mo (HR 0.897 95% CI 0.623–1.294) (67)
PALOMA-2	III	First line	Palbociclib/Letrozole vs. Letrozole	666	27.6mo vs. 14.5mo (HR 0.563; 0.461-0.687 p<0.0001) (1)	Pending
PALOMA-3	III	Second line	Palbociclib/Fulvestrant vs. Fulvestrant	521	9.5 months vs. 4.6 months (HR 0·46, 0·36–0·59, p<0·0001) (68)	Not significant 34.9 months vs. 28 months (HR 0.81; 95% CI 0.64 – 1.03) (3)
**Ribociclib**						
MONALEESA-2	III	First line	Ribociclib/Letrozole vs. Letrozole	668	25.3 months versus 16 months (HR 0.568; 0.457–0.704; P = 9.63 × 10−8) (69)	Not reached vs. 33 mo (HR 0.746; CI 0.517-1.078) (69)
MONALEESA-3	III	First and 2nd line	Ribociclib/Fulvestrant vs. Fulvestrant	725	20.5 months versus 12.8 months (HR 0.593; 0.480 to 0.732; P < .001)	Statistically significant Not reached vs. 40.0 mo (HR=0.74; 95% CI, 0.568-0.924; P=.00455) (70)
MONALEESA-7	III	First line	Ribociclib/OFS/AI or tamoxifen vs. OFS/AI or tamoxifen	672	23.8 months versus 13 months (HR 0·55; 0·44–0·69; p<0·0001) (6)	Statistically significant Not Reached vs 40.9 mo (HR, 0.712, 0.54-0.95; p = 0.00973) (17)
**Abemaciclib**						
MONARCH-1	II	Later lines	Abemaciclib	132	6 months (58)	17.7 mo (58)
MONARCH-2	III	Second line	Abemaciclib/Fulvestrant vs. Fulvestrant	669	16.4 months versus 9.3 months (HR 0.553; 0.449 to 0.681; P < .001) (7)	Statistically significant. 46.7 vs. 37.3 mo (HR=0.757; 95% CI, 0.606- 0.945; P=.0137) (71)
MONARCH-3	III	First line	Abemaciclib/AI vs. Abemaciclib	493	28.18 versus 14.76 months; (HR 0.540; 0.418–0.698; p = .000002) (5)	Pending

### CNS-Specific Efficacy

Patients with CNS metastases from breast cancer often have limited treatment options due to poor penetration of the blood-brain-barrier (BBB) by most systemic agents. Pre-clinical models have suggested that palbociclib and ribociclib may have poor CNS penetration while abemaciclib may be able to cross the BBB effectively.

Abemaciclib was noted to cross the BBB in xenograft models (35). In a study that looked at the penetration of abemaciclib and palbociclib in mouse xenograft models across the BBB, abemaciclib was found to have better penetration into the central nervous system compared to palbociclib (72). This is most likely due to the strong efflux of palbociclib out of the central nervous system compared to abemaciclib (72). The cerebrospinal fluid concentration of abemaciclib ranged from 2.2 to 14.7 nmol/L, which was beyond the dissociation constant of CDK4/cyclin D1 combination and was close to the unbound plasma concentrations (57).

A common mechanism by which drug penetration of the BBB is prevented is *via* the ATP-binding cassette (ABC) efflux transporters. Endothelial cells that form the BBB limit passage of solutes into the brain except by passive diffusion or uptake transport. This barrier is also fortified by efflux pumps which actively transport substrates out of the brain. Such transporters include P-glycoprotein (P-gp) and breast cancer resistance protein (BCRP) (73).

In vitro transwell assays have shown that palbociclib is a substrate of both P-gp and BCRP, and that ribociclib is a substrate of P-gp, restricting their ability to penetrate the BBB (74, 75).

In contrast, abemaciclib is both a substrate and an inhibitor of P-gp and BRCP substrate, therefore, the efficiency of efflux has been shown to be less than that seen with palbociclib (72). Tolaney et al. reported a phase II non-randomized clinical trial which evaluated the role of abemaciclib in patients with brain or leptomeningeal metastases (LM) secondary to HR+ metastatic breast cancer (76). The study showed intracranial clinical benefit rate of 24% in heavily pretreated HR+, HER2− patient cohort (76). Patients with leptomeningeal disease was noted to have median PFS of 5.9 months. Abemaciclib and its active metabolites were noted in brain metastases tissue (76). This is one of the first studies to show CNS benefit with abemacicib in heavily pre-treated patients.

### Biomarkers Implicated in Resistance Mechanisms

Despite significant clinical activity of these inhibitors, both primary resistance and development of acquired resistance can occur. There is a great need to develop biomarkers of sensitivity and resistance to these agents. Multiple potential mechanisms of resistance to CDK4/6 inhibitors have been identified, including loss of RB, elevated CDK6 activity, FGFR pathway activation and Cyclin E-CDK2 activation.

Given that the primary target of CDK4/6 is RB1, loss of RB1 function, is predictive of shorter recurrence free survival times (77). Palbociclib did not have activity in cancer cells that had deletion or inactivation of RB (78–80). RB-deficient tumors tend to demonstrate extremely high expression of p16INK4A (81). In a study that looked at the activity of palbociclib in explanted breast cancers, p16ink4a-high tumors were also noted to be unresponsive to palbociclib (80).

Cell lines developed to acquire resistance to Palbociclib (through chronic exposure to the drug), have been shown to have loss of Rb expression and over-expression of Cyclin E1 (82). In-vitro studies have shown cells adapting to CDK4/6 inhibition with palbociclib or ribociclib *via* phosphorylation of Rb within 48 hours, thus allowing treated cells to enter the S-phase (40, 82). However, with abemaciclib, cells were shown to go for as long as 5 days without evidence of cell adaptation (40). Ribociclib resistant cell lines were resistant to abemaciclib as well and vice versa (83). Abemaciclib resistant cell lines were noted to have CDK6 upregulation, which was not seen in ribociclib resistant cell lines (83).

RBsig, a gene expression signature of Rb loss-of-function, has been associated with sensitivity to abemaciclib monotherapy in tumors derived from the neoMONARCH study (84). Tumors resistant to abemaciclib were noted to have higher expression of Cyclin E1 and Rb loss of function score, compared to abemaciclib sensitive tumors, however this was not statistically significant (85).

Due to abemaciclib’s ability to inhibit other CDKs in addition to CDK4/6, it has been shown to be effective in inhibiting cell growth even in RB deficient cell lines which are resistant to palbociclib and ribociclib (40, 44). RB-deficient cells treated with ribociclib or palbociclib had no effect on cell-cycle distribution, however treatment with abemaciclib caused cells to accumulate in G2, consistent with an abemaciclib-induced cell cycle arrest independent of CDK4/6 (40).

Cyclin E is a regulatory subunit of CDK2 which induces transition into S-phase, and it is degraded as cells progress through S phase. CDK2 is not a target of either palbociclib or ribociclib. Cyclin E overexpression renders the cells ineffective to CDK4/6 inhibition and G1 arrest. Tumor tissue analysis from patients enrolled in the PALOMA-3 trial demonstrated that patients with higher levels of Cyclin E mRNA expression were less susceptible to palbociclib. They had significantly shorter progression free survival times than tumors with low Cyclin E expression (86). Western blot analysis of palbociclib resistant cells lines showed increase in Cyclin E and phosphorylated CDK2 and were shown to be only partially resistant to abemaciclib (87).

Inhibition of CDK2/Cyclin E circumvents resistance resulting from Cyclin E amplification, which is a mechanism that has been identified in palbociclib-resistant cell lines. Abemaciclib inhibits cyclin E in preclinical cell lines. Therefore, patients whose tumors have high levels of Cyclin E expression, and who have progressed or shown no response to palbociclib, may derive some benefit from subsequent treatment with abemaciclib. Haffner et al. reported the case of a 75 year old woman with ER+/PR+/HER2 negative metastatic breast cancer who had initial response to combination therapy with fulvestrant and palbociclib (40) She then developed progression of disease with liver metastases and was switched to single agent abemaciclib (200 mg twice a day) which resulted in the decrease of the size of the liver lesion within three months of monotherapy (40). Treatment with abemaciclib maybe an option in a sub-set of patients who progress on palbociclib.

Upregulation of FGFR signaling has been identified as a mechanism of drug resistance to both ribociclib and palbociclib. Circulating tumor DNA obtained in patients enrolled in the MONALEESA-2 trial of ribociclib showed a shorter progression free survival in patients with an FGFR1 amplification compared to those with wild type FGFR1 (88). FGFR1 is part of the 8p11 amplicon, and 8p11 amplification is a known predictor of poor response to hormonal therapy in ER+ breast cancers (89). In MONALEESA-3, the PFS in tumors with FGFR1 amplification in the ribociclib plus fulvestrant arm compared to placebo plus fulvestrant arm was not statistically significant. (10.97 months vs 6.67 months HR 0.73; CI 0.37-1.43) (90). In contrast, in MONARCH 3, there was an improvement in PFS in tumors with FGFR1 mutation amplification treated with abemaciclib and aromatase inhibitors compared to aromatase inhibitors alone (32.8 months vs. 7.6 months HR 0.37; 95% CI 0.16 to 0.85) (91). This suggests that FGFR1 amplification may be a marker of resistance to palbociclib and ribociclib, but not to abemaciclib. Further studies will be needed to validate these findings.

The mechanisms of resistances between the three approved CDK4/6 inhibitors are dissimilar and more work needs to be done to understand the unique resistance mechanisms for these agents. Identification of *de novo* biomarkers of resistance would allow improved patient selection.

## Conclusion

The approved CDK4/6 inhibitors currently on the market have been shown to improve PFS and OS when used in combination with hormonal therapy, in patients with HR+/HER2 negative metastatic breast cancer. Intriguingly only abemaciclib, the least specific inhibitor, has data supporting a role in the adjuvant setting and is the only CDK4/6 inhibitor approved as monotherapy in the metastatic setting. Although they all inhibit CDK4/6, there are differences in target specificity, dosing schedules, CNS penetration and toxicity between these agents that prescribers may need to take into consideration before initiating therapy.

Further work needs to be done to better understand unique tissue and/or serum biomarkers that may predict benefit for each of the approved CDK4/6 inhibitors to guide patient selection and optimal treatment combinations. New data showing effect of the CDK4/6 inhibitors on immune signaling, if validated, may broaden the potential utility of these inhibitors in cancer therapy.

## Author Contributions

All authors listed have made a substantial, direct, and intellectual contribution to the work, and approved it for publication.

## Conflict of Interest

The authors declare that the research was conducted in the absence of any commercial or financial relationships that could be construed as a potential conflict of interest.
